# 1,3,4-Tri-*O*-acetyl-2-*N*-(tri­fluoro­acetyl)-β-l-fucose

**DOI:** 10.1107/S1600536813034958

**Published:** 2014-01-15

**Authors:** David C. McCutcheon, Peter Norris, Matthias Zeller

**Affiliations:** a Department of Chemistry, Youngstown State University, 1 University Plaza, Youngstown, OH 44555-3663, USA

## Abstract

The title compound, C_14_H_18_F_3_NO_8_, was produced through conjugation of 1,3,4-tri-*O*-acetyl-2-azidode­oxy-α,β-l-fucose with tri­fluoro­acetyl chloride in the presence of bis­(di­phenyl­phosphino)ethane in tetra­hydro­furan at room temperature. The X-ray crystal structure reveals that the β-anomer of the product mixture crystallizes from ethyl acetate/hexa­nes. The compound exists in a typical chair conformation with the maximum possible number of substituents, four out of five, located in the sterically preferred equatorial positions. The major directional force facilitating packing of the mol­ecules are N—H⋯O hydrogen bonds involving the amide moieties of neighboring mol­ecules, which connect mol­ecules stacked along the *a*-axis direction into infinite strands with a *C* ^1^_1_(4) graph-set motif. Formation of the strands is assisted by a number of weaker C—H⋯O inter­actions involving the methine and methyl H atoms. These strands are connected through further C—H⋯O and C—H⋯F inter­actions into a three dimensional network

## Related literature   

Information related to the synthesis of *N*-acetyl-l-fucosa­mine analogues may be found in Alhassan *et al.* (2012 ▶). Rao *et al.* (1998 ▶) describe conformations of carbohydrate mol­ecules.
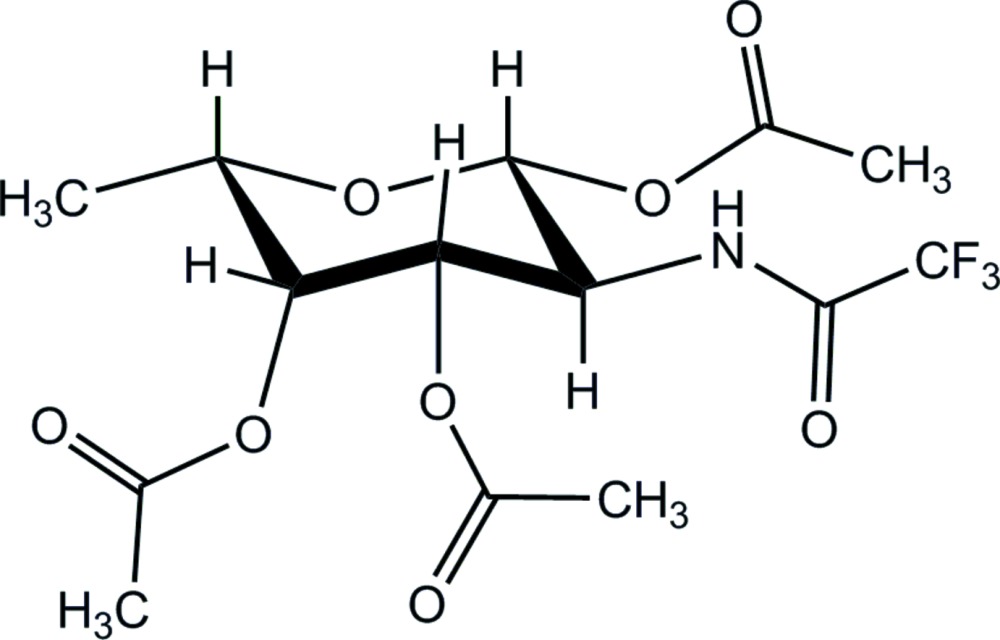


## Experimental   

### 

#### Crystal data  

C_14_H_18_F_3_NO_8_
*M* *_r_* = 385.29
Orthorhombic, 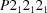

*a* = 5.1818 (10) Å
*b* = 16.968 (3) Å
*c* = 19.484 (4) Å
*V* = 1713.1 (6) Å^3^
*Z* = 4
Mo *K*α radiation
μ = 0.14 mm^−1^
*T* = 100 K
0.48 × 0.31 × 0.30 mm

#### Data collection  

Bruker AXS Smart Apex CCD diffractometer
Absorption correction: multi-scan (*SADABS*; Sheldrick, 2007 ▶) *T*_min_ = 0.839, *T*_max_ = 0.958
11394 measured reflections
4232 independent reflections
3869 reflections with *I* > 2σ(*I*)
*R*_int_ = 0.029

#### Refinement  

*R*[*F* ^2^ > 2σ(*F* ^2^)] = 0.037
*wR*(*F* ^2^) = 0.088
*S* = 1.06
4232 reflections
239 parameters
H-atom parameters constrained
Δρ_max_ = 0.30 e Å^−3^
Δρ_min_ = −0.17 e Å^−3^
Absolute structure: Flack parameter determined using 1493 quotients [(*I* ^+^)−(*I* ^−^)]/[(*I* ^+^)+(*I* ^−^)] (Parsons *et al.*, 2013 ▶)
Absolute structure parameter: 0.4 (3)

### 

Data collection: *SMART* (Bruker, 2002 ▶); cell refinement: *SAINT-Plus* (Bruker, 2003 ▶); data reduction: *SAINT-Plus*; program(s) used to solve structure: *SHELXS97* (Sheldrick, 2008 ▶); program(s) used to refine structure: *SHELXL2013* (Sheldrick, 2008 ▶) and *SHELXLE* (Hübschle *et al.*, 2011 ▶); molecular graphics: *Mercury* (Macrae *et al.*, 2008 ▶); software used to prepare material for publication: *publCIF* (Westrip, 2010 ▶).

## Supplementary Material

Crystal structure: contains datablock(s) I, global. DOI: 10.1107/S1600536813034958/bv2229sup1.cif

Structure factors: contains datablock(s) I. DOI: 10.1107/S1600536813034958/bv2229Isup2.hkl

CCDC reference:

Additional supporting information:  crystallographic information; 3D view; checkCIF report

## Figures and Tables

**Table 1:** Hydrogen-bond geometry (Å, °)

*D*—H⋯*A*	*D*—H	H⋯*A*	*D*⋯*A*	*D*—H⋯*A*
N1—H1*A*⋯O6^i^	0.88	2.14	2.959 (2)	155
C3—H3⋯O6^i^	1.00	2.45	3.308 (3)	144
C6—H6*B*⋯F3^ii^	0.98	2.63	3.487 (3)	147
C8—H8*A*⋯O5^iii^	0.98	2.48	3.445 (3)	169
C8—H8*B*⋯O5^iv^	0.98	2.41	3.206 (3)	137
C8—H8*C*⋯O8^v^	0.98	2.64	3.443 (3)	139
C14—H14*A*⋯O8^iv^	0.98	2.38	3.292 (3)	155

Symmetry codes: (i) 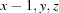; (ii) 
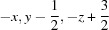
; (iii) 
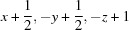
; (iv) 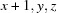; (v) 
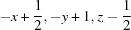
.

**Fig. 1.**
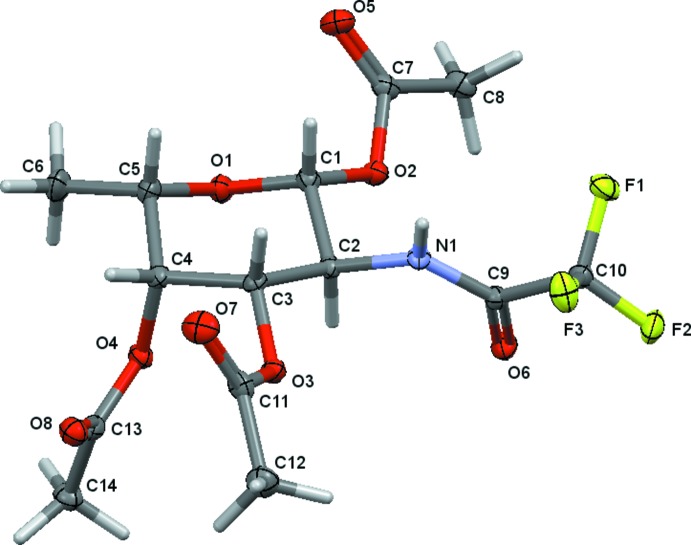
: Thermal ellipsoid plot, with atom labels for non-hydrogen atoms and 50 percent probability ellipsoids.

**Fig. 2.**
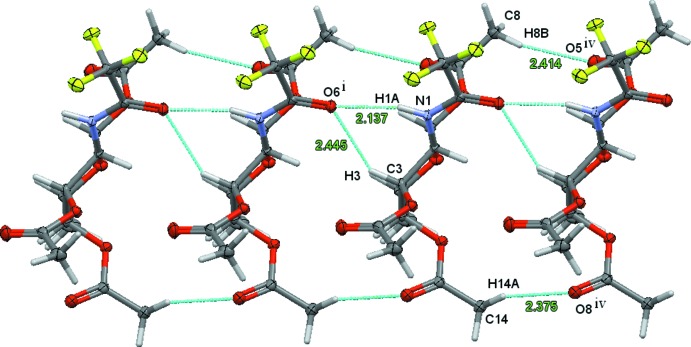
: Major packing interactions, viewed roughly perpendicular to the a axis direction showing the strands parallel to [1 0 0]. Blue dotted lines denote N—H···O hydrogen bonds and assisting C—H···O interactions. Symmetry codes: (i) x - 1, y, z; (iv) x + 1, y, z.

**Fig. 3.**
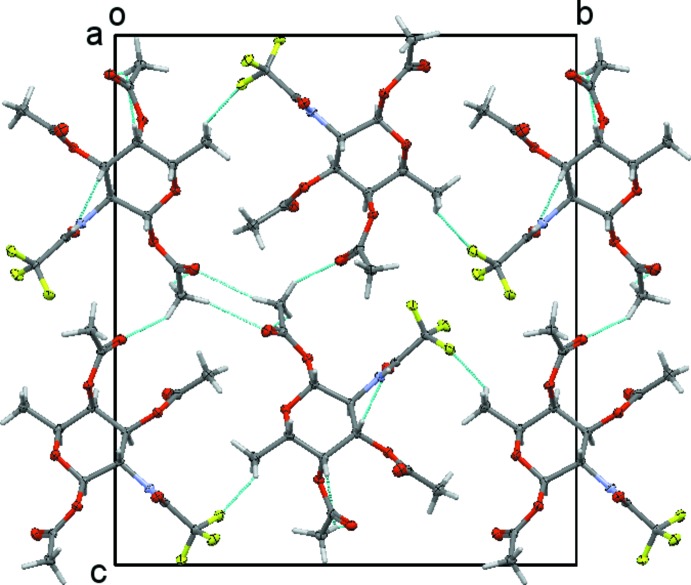
: View along the a axis direction, showing C—H···O and C—H···F interactions connecting parallel strands with each other. For atoms involved and symmetry operators, see Table 1.

## supplementary crystallographic information

### 1. Comment

The title compound, 1,3,4-tri-*O*-acetyl-2-*N*-(trifluoro)acetyl-β-*L*-fucose, was synthesized during a project focused on the production of glycomimetic analogs of the bacterial aminosugar, *N*-acetyl-*L*-fucosamine (Alhassan *et al.*, 2012). Beginning with an anomeric mixture of 1,3,4-tri-*O*-acetyl-2-azidodeoxy-α,β-*L*-fucoses, treatment with trifluoroacetyl chloride in the presence of bis(diphenylphosphino)ethane in THF at room temperature afforded a mixture of 1,3,4-tri-*O*-acetyl-2-*N*-(trifluoro)acetyl-α,β-*L*-fucoses as a colorless syrup. The mixture of anomers was purified by column chromatography, and one of the two anomers was selectively isolated by vapor diffusion crystallization using ethyl acetate and hexanes, with the other anomer remaining in solution. The crystals (m.p. 157–159 °C) were initially identified as the β-anomer by ^1^H NMR spectroscopy. Single-crystal diffraction was then employed in order to unambiguously confirm the configuration of the anomer isolated by crystallization.

Figure 1 shows a depiction of one molecule of 1,3,4-tri-*O*-acetyl-2-*N*-(trifluoro)acetyl-β-*L*-fucose as present in the solid state. From the crystal structure, it was confirmed that the isolated and crystallized fraction is indeed the β anomer, as had been already suspected based on ^1^H NMR shifts and coupling constants. The molecule crystallizes in a chair conformation as typical for pyranose sugar derivatives (Rao *et al.*, 1998) with the choice of the chair conformation – from the two that are possible – being apparently the result of sterical interactions. The maximum possible number of substituents, four out of five, are located in the sterically preferred equatorial positions. Only the *O*-acetyl group at carbon atom C4 is, out of necessity to be able to maintain the overall chair conformation, forced into an axial position. This conformation is in agreement with the observed solution conformation as determined from the ^1^H NMR coupling constants (see experimental section). The acetyl and amide groups are, as expected, nearly perfectly planar – the r.m.s. deviation from planarity for the five atoms of the amide group is only 0.0052. Those for the four atoms of each acetate group at C1, C3 and C4 are 0.0196, 0.0087 and 0.0475, respectively – and they are tilted to variable degrees against the average plane of the pyranose moiety (48.17 (9)° at C1, 57.98 (9)° at C3, 79.04 (7)° at C4, and 71.97 (8)° for the amide group).

This conformation allows for dense packing of the molecules in the solid state, with no significant distortions of the molecules or voids present in the solid state. The major directional force facilitating packing of the molecules are N—H···O hydrogen bonds involving the amide moieties of neighboring molecules. Through these interactions, molecules stacked along the direction of the *a*-axis (created through translation from each other) are connected into infinite strands, with a C^1^_1_(4) graph set motif for the N—H···O hydrogen bonds. Formation of the strands is assisted by a number of weaker but nevertheless still attractive C—H···O interactions involving the methine and methyl hydrogen atoms, with three of these interactions featuring H···O separations of 2.5 Å or less (2.38, 2.41 and 2.45 Å, involving H14A, H8B and H3, respectively. See Table 1 for details and symmetry operators). These strands, Figure 2, are in turn connected with each other through further C—H···O and C—H···F interactions into a three dimensional network, Figure 3 and Table 1. The stabilizing and directional effect of the C—H···F interactions might also have contributed to the ordered nature of the trifluoro methyl group. No signs of rotational disorder, as often observed for CF_3_ groups, is evident for this structure.

### 2. Experimental

The title compound was synthesized from 1,3,4-tri-*O*-acetyl-2-azidodeoxy-α,β-*L*-fucoses (anomeric α/β ratio of 3:2 by NMR) by Staudinger-type synthesis with trifluoroacetyl chloride in the presence of bis(diphenylphosphino)ethane following a previously reported procedure (Alhassan *et al.*, 2012). Column chromatography (silica gel, 2:1 hexanes-EtOAc) yielded a 3:2 anomeric α/β mixture (by NMR) of the title compound as a colorless syrup in an overall yield of 37%. The β-anomer was selectively isolated as a crystalline solid by vapor diffusion from ethyl acetate and hexanes, m.p. 157–159 °C.

^1^H NMR (400 MHz, CDC1_3_): 1.25 (d, 3H, H-6, *J* = 6.40 Hz); 2.02 (s, 3H, COCH_3_); 2.12 (s, 3H, COCH_3_); 2.21 (s, 3H, COCH_3_); 3.94 (dq, 1H, H-5, *J* = 0.96, 6.44 Hz); 4.47 (ddd, 1H, H-2, *J* = 9.28, 9.28, 11.12 Hz); 5.14 (dd, 1H, H-3, *J* = 3.28, 11.28 Hz); 5.25 (dd, 1H, H-4, *J* = 0.78, 3.30 Hz); 5.74 (d, 1H, H-1, *J* = 8.76 Hz); 6.56 (d, 1H, N—H, *J* = 9.44 Hz).

^13^C NMR (100 MHz, CDC1_3_): 16.03 (C-6); 20.45 (COCH_3_); 20.63 (COCH_3_); 20.68 (COCH_3_); 50.48 (C-2); 69.18 (C-4); 70.28 (C-3); 70.75 (C-5); 92.40 (C-1); 157.44 (COCF_3_); 157.81 (COCF_3_); 169.64 (COCH_3_); 170.43 (COCH_3_); 170.90 (COCH_3_).

MS: *m/z* calculated: 385.1 *m/z* found (ESI): 408.2 (+Na+).

### 3. Refinement

H atoms attached to carbon and nitrogen atoms were positioned geometrically and constrained to ride on their parent atoms, with carbon hydrogen bond distances of 1.00 and 0.98 Å for C—H and CH_3_ and 0.88 Å for N—H moieties, respectively. Methyl H atoms were allowed to rotate but not to tip to best fit the experimental electron density. *U*_iso_(H) values were set to a multiple of *U*_eq_(C/N) with 1.5 for CH_3_ and 1.2 for C—H and N—H units, respectively. Reflection 0 1 1 was affected by the beam stop and was omitted from the refinement.

### Crystal data

**Table:**

C_14_H_18_F_3_NO_8_	*D*_x_ = 1.494 Mg m^−^^3^
*M**_r_* = 385.29	Melting point = 430.5–432.5 K
Orthorhombic, *P*2_1_2_1_2_1_	Mo *K*α radiation, λ = 0.71073 Å
Hall symbol: P 2ac 2ab	Cell parameters from 6913 reflections
*a* = 5.1818 (10) Å	θ = 2.4–30.5°
*b* = 16.968 (3) Å	µ = 0.14 mm^−^^1^
*c* = 19.484 (4) Å	*T* = 100 K
*V* = 1713.1 (6) Å^3^	Block, colourless
*Z* = 4	0.48 × 0.31 × 0.30 mm
*F*(000) = 800	

### Data collection

**Table:**

Bruker AXS Smart Apex CCD diffractometer	4232 independent reflections
Radiation source: fine-focus sealed tube	3869 reflections with *I* > 2σ(*I*)
Graphite monochromator	*R*_int_ = 0.029
ω scans	θ_max_ = 28.3°, θ_min_ = 2.1°
Absorption correction: multi-scan (*SADABS*; Sheldrick, 2007)	*h* = −6→6
*T*_min_ = 0.839, *T*_max_ = 0.958	*k* = −22→21
11394 measured reflections	*l* = −22→25

### Refinement

**Table:**

Refinement on *F*^2^	Secondary atom site location: difference Fourier map
Least-squares matrix: full	Hydrogen site location: inferred from neighbouring sites
*R*[*F*^2^ > 2σ(*F*^2^)] = 0.037	H-atom parameters constrained
*wR*(*F*^2^) = 0.088	*w* = 1/[σ^2^(*F*_o_^2^) + (0.0424*P*)^2^ + 0.1715*P*] where *P* = (*F*_o_^2^ + 2*F*_c_^2^)/3
*S* = 1.06	(Δ/σ)_max_ < 0.001
4232 reflections	Δρ_max_ = 0.30 e Å^−^^3^
239 parameters	Δρ_min_ = −0.17 e Å^−^^3^
0 restraints	Absolute structure: Flack parameter determined using 1493 quotients [(*I*^+^)-(*I*^-^)]/[(*I*^+^)+(*I*^-^)] (Parsons *et al.*, 2013)
Primary atom site location: structure-invariant direct methods	Absolute structure parameter: 0.4 (3)

### Special details

**Table:**

Geometry. All e.s.d.'s (except the e.s.d. in the dihedral angle between two l.s. planes) are estimated using the full covariance matrix. The cell e.s.d.'s are taken into account individually in the estimation of e.s.d.'s in distances, angles and torsion angles; correlations between e.s.d.'s in cell parameters are only used when they are defined by crystal symmetry. An approximate (isotropic) treatment of cell e.s.d.'s is used for estimating e.s.d.'s involving l.s. planes.

### Fractional atomic coordinates and isotropic or equivalent isotropic displacement parameters (Å^2^)

**Table:**

	*x*	*y*	*z*	*U*_iso_*/*U*_eq_	
C1	0.3282 (4)	0.43097 (12)	0.65855 (10)	0.0225 (4)	
H1	0.1566	0.4327	0.6351	0.027*	
C2	0.3854 (4)	0.50942 (11)	0.69399 (10)	0.0190 (4)	
H2	0.5626	0.5077	0.7143	0.023*	
C3	0.1889 (4)	0.52308 (11)	0.75081 (10)	0.0209 (4)	
H3	0.0174	0.5351	0.7297	0.025*	
C4	0.1619 (4)	0.45132 (12)	0.79776 (11)	0.0239 (4)	
H4	0.0089	0.4585	0.8284	0.029*	
C5	0.1274 (5)	0.37669 (12)	0.75536 (11)	0.0284 (5)	
H5	−0.0392	0.3801	0.7296	0.034*	
C6	0.1278 (6)	0.30211 (14)	0.79839 (12)	0.0417 (7)	
H6A	0.1026	0.2563	0.7685	0.063*	
H6B	−0.0124	0.3046	0.8321	0.063*	
H6C	0.2934	0.2973	0.8223	0.063*	
C7	0.4717 (4)	0.36394 (12)	0.55954 (11)	0.0255 (5)	
C8	0.6856 (5)	0.35718 (14)	0.50908 (11)	0.0306 (5)	
H8A	0.7102	0.3017	0.4968	0.046*	
H8B	0.8448	0.3779	0.5294	0.046*	
H8C	0.6427	0.3875	0.4678	0.046*	
C9	0.5799 (4)	0.60760 (12)	0.61779 (10)	0.0201 (4)	
C10	0.5121 (4)	0.67368 (12)	0.56627 (11)	0.0231 (4)	
C11	0.0942 (5)	0.63360 (13)	0.82140 (11)	0.0274 (5)	
C12	0.2160 (6)	0.70111 (14)	0.85856 (13)	0.0361 (6)	
H12A	0.0826	0.7392	0.8716	0.054*	
H12B	0.3424	0.7268	0.8285	0.054*	
H12C	0.3029	0.6816	0.8999	0.054*	
C13	0.3851 (4)	0.47114 (12)	0.90408 (11)	0.0247 (4)	
C14	0.6170 (4)	0.44563 (14)	0.94350 (11)	0.0306 (5)	
H14A	0.7671	0.4764	0.9287	0.046*	
H14B	0.6492	0.3895	0.9351	0.046*	
H14C	0.5873	0.4542	0.9926	0.046*	
F1	0.4052 (3)	0.64366 (8)	0.50988 (6)	0.0343 (3)	
F2	0.7207 (3)	0.71321 (8)	0.54797 (7)	0.0374 (3)	
F3	0.3429 (3)	0.72485 (8)	0.59280 (7)	0.0373 (4)	
N1	0.3700 (3)	0.57409 (10)	0.64480 (8)	0.0203 (4)	
H1A	0.2170	0.5916	0.6325	0.024*	
O1	0.3363 (3)	0.36940 (8)	0.70706 (7)	0.0269 (3)	
O2	0.5281 (3)	0.41733 (8)	0.61054 (7)	0.0236 (3)	
O3	0.2779 (3)	0.59116 (8)	0.78734 (7)	0.0230 (3)	
O4	0.3917 (3)	0.44154 (8)	0.83929 (7)	0.0241 (3)	
O5	0.2690 (3)	0.32900 (9)	0.55792 (9)	0.0338 (4)	
O6	0.8048 (3)	0.59089 (9)	0.62873 (8)	0.0272 (3)	
O7	−0.1313 (3)	0.61666 (11)	0.82064 (9)	0.0382 (4)	
O8	0.2109 (3)	0.51128 (9)	0.92552 (8)	0.0292 (4)	

### Atomic displacement parameters (Å^2^)

**Table:**

	*U*^11^	*U*^22^	*U*^33^	*U*^12^	*U*^13^	*U*^23^
C1	0.0229 (11)	0.0235 (9)	0.0210 (10)	0.0005 (8)	−0.0013 (8)	0.0021 (8)
C2	0.0170 (9)	0.0221 (9)	0.0181 (9)	−0.0001 (8)	−0.0011 (8)	0.0034 (7)
C3	0.0173 (9)	0.0235 (9)	0.0220 (10)	−0.0017 (8)	−0.0006 (8)	0.0003 (8)
C4	0.0200 (10)	0.0290 (10)	0.0228 (10)	−0.0037 (9)	−0.0008 (8)	0.0028 (8)
C5	0.0312 (12)	0.0276 (10)	0.0265 (11)	−0.0090 (9)	−0.0003 (10)	0.0044 (8)
C6	0.0604 (18)	0.0305 (12)	0.0342 (13)	−0.0151 (13)	0.0019 (13)	0.0073 (10)
C7	0.0296 (12)	0.0216 (10)	0.0251 (11)	0.0053 (9)	−0.0062 (9)	0.0005 (8)
C8	0.0313 (12)	0.0330 (11)	0.0275 (11)	0.0063 (10)	−0.0004 (10)	−0.0045 (9)
C9	0.0193 (10)	0.0229 (9)	0.0181 (9)	−0.0008 (8)	0.0011 (8)	−0.0001 (7)
C10	0.0237 (10)	0.0225 (10)	0.0233 (10)	−0.0010 (8)	0.0014 (8)	−0.0006 (8)
C11	0.0282 (12)	0.0292 (11)	0.0247 (11)	0.0061 (9)	0.0019 (9)	0.0026 (8)
C12	0.0418 (15)	0.0329 (12)	0.0335 (12)	0.0019 (11)	0.0022 (11)	−0.0054 (10)
C13	0.0235 (11)	0.0270 (10)	0.0237 (10)	−0.0045 (9)	0.0001 (9)	0.0037 (8)
C14	0.0259 (11)	0.0385 (12)	0.0275 (11)	0.0003 (10)	−0.0032 (10)	0.0007 (9)
F1	0.0460 (9)	0.0312 (6)	0.0258 (7)	0.0004 (6)	−0.0113 (6)	0.0026 (5)
F2	0.0354 (8)	0.0380 (8)	0.0389 (8)	−0.0104 (6)	0.0042 (7)	0.0133 (6)
F3	0.0483 (9)	0.0295 (6)	0.0342 (7)	0.0157 (7)	0.0119 (7)	0.0063 (5)
N1	0.0159 (8)	0.0237 (8)	0.0214 (8)	0.0009 (7)	−0.0009 (7)	0.0041 (6)
O1	0.0334 (9)	0.0230 (7)	0.0243 (7)	−0.0011 (7)	−0.0003 (7)	0.0038 (6)
O2	0.0216 (7)	0.0260 (7)	0.0233 (7)	0.0006 (6)	−0.0002 (6)	−0.0022 (6)
O3	0.0210 (7)	0.0241 (7)	0.0240 (7)	−0.0021 (6)	0.0018 (6)	−0.0021 (6)
O4	0.0231 (8)	0.0286 (7)	0.0206 (7)	0.0004 (6)	−0.0021 (6)	0.0029 (6)
O5	0.0306 (9)	0.0324 (9)	0.0385 (9)	−0.0035 (7)	−0.0044 (7)	−0.0076 (7)
O6	0.0177 (7)	0.0354 (8)	0.0286 (8)	−0.0002 (6)	−0.0004 (6)	0.0061 (6)
O7	0.0234 (9)	0.0477 (10)	0.0435 (10)	0.0088 (8)	0.0017 (8)	−0.0075 (8)
O8	0.0268 (8)	0.0343 (8)	0.0265 (8)	0.0018 (7)	0.0003 (7)	−0.0004 (6)

### Geometric parameters (Å, º)

**Table:**

C1—O1	1.410 (2)	C8—H8A	0.9800
C1—O2	1.415 (3)	C8—H8B	0.9800
C1—C2	1.528 (3)	C8—H8C	0.9800
C1—H1	1.0000	C9—O6	1.218 (3)
C2—N1	1.459 (2)	C9—N1	1.335 (3)
C2—C3	1.522 (3)	C9—C10	1.545 (3)
C2—H2	1.0000	C10—F2	1.321 (2)
C3—O3	1.433 (2)	C10—F1	1.332 (2)
C3—C4	1.529 (3)	C10—F3	1.338 (2)
C3—H3	1.0000	C11—O7	1.203 (3)
C4—O4	1.449 (3)	C11—O3	1.366 (3)
C4—C5	1.523 (3)	C11—C12	1.495 (3)
C4—H4	1.0000	C12—H12A	0.9800
C5—O1	1.440 (3)	C12—H12B	0.9800
C5—C6	1.518 (3)	C12—H12C	0.9800
C5—H5	1.0000	C13—O8	1.205 (3)
C6—H6A	0.9800	C13—O4	1.359 (3)
C6—H6B	0.9800	C13—C14	1.490 (3)
C6—H6C	0.9800	C14—H14A	0.9800
C7—O5	1.207 (3)	C14—H14B	0.9800
C7—O2	1.376 (3)	C14—H14C	0.9800
C7—C8	1.486 (3)	N1—H1A	0.8800
			
O1—C1—O2	107.48 (16)	C7—C8—H8B	109.5
O1—C1—C2	109.69 (16)	H8A—C8—H8B	109.5
O2—C1—C2	107.42 (17)	C7—C8—H8C	109.5
O1—C1—H1	110.7	H8A—C8—H8C	109.5
O2—C1—H1	110.7	H8B—C8—H8C	109.5
C2—C1—H1	110.7	O6—C9—N1	127.7 (2)
N1—C2—C3	109.07 (16)	O6—C9—C10	120.00 (18)
N1—C2—C1	110.34 (15)	N1—C9—C10	112.33 (17)
C3—C2—C1	109.37 (16)	F2—C10—F1	108.16 (17)
N1—C2—H2	109.3	F2—C10—F3	108.13 (16)
C3—C2—H2	109.3	F1—C10—F3	107.12 (18)
C1—C2—H2	109.3	F2—C10—C9	110.97 (18)
O3—C3—C2	105.59 (16)	F1—C10—C9	110.66 (16)
O3—C3—C4	111.98 (16)	F3—C10—C9	111.64 (17)
C2—C3—C4	112.03 (17)	O7—C11—O3	123.0 (2)
O3—C3—H3	109.0	O7—C11—C12	126.8 (2)
C2—C3—H3	109.0	O3—C11—C12	110.2 (2)
C4—C3—H3	109.0	C11—C12—H12A	109.5
O4—C4—C5	107.73 (17)	C11—C12—H12B	109.5
O4—C4—C3	110.49 (17)	H12A—C12—H12B	109.5
C5—C4—C3	110.39 (16)	C11—C12—H12C	109.5
O4—C4—H4	109.4	H12A—C12—H12C	109.5
C5—C4—H4	109.4	H12B—C12—H12C	109.5
C3—C4—H4	109.4	O8—C13—O4	123.3 (2)
O1—C5—C6	106.8 (2)	O8—C13—C14	126.1 (2)
O1—C5—C4	109.73 (17)	O4—C13—C14	110.56 (19)
C6—C5—C4	113.17 (18)	C13—C14—H14A	109.5
O1—C5—H5	109.0	C13—C14—H14B	109.5
C6—C5—H5	109.0	H14A—C14—H14B	109.5
C4—C5—H5	109.0	C13—C14—H14C	109.5
C5—C6—H6A	109.5	H14A—C14—H14C	109.5
C5—C6—H6B	109.5	H14B—C14—H14C	109.5
H6A—C6—H6B	109.5	C9—N1—C2	122.31 (17)
C5—C6—H6C	109.5	C9—N1—H1A	118.8
H6A—C6—H6C	109.5	C2—N1—H1A	118.8
H6B—C6—H6C	109.5	C1—O1—C5	110.62 (16)
O5—C7—O2	121.8 (2)	C7—O2—C1	115.46 (16)
O5—C7—C8	126.4 (2)	C11—O3—C3	116.22 (17)
O2—C7—C8	111.75 (19)	C13—O4—C4	117.13 (17)
C7—C8—H8A	109.5		
			
O1—C1—C2—N1	−178.28 (17)	N1—C9—C10—F3	50.2 (2)
O2—C1—C2—N1	65.2 (2)	O6—C9—N1—C2	0.2 (3)
O1—C1—C2—C3	−58.3 (2)	C10—C9—N1—C2	179.05 (16)
O2—C1—C2—C3	−174.82 (16)	C3—C2—N1—C9	137.83 (19)
N1—C2—C3—O3	−67.37 (19)	C1—C2—N1—C9	−102.0 (2)
C1—C2—C3—O3	171.87 (15)	O2—C1—O1—C5	−176.42 (15)
N1—C2—C3—C4	170.49 (16)	C2—C1—O1—C5	67.1 (2)
C1—C2—C3—C4	49.7 (2)	C6—C5—O1—C1	171.70 (18)
O3—C3—C4—O4	−48.1 (2)	C4—C5—O1—C1	−65.3 (2)
C2—C3—C4—O4	70.4 (2)	O5—C7—O2—C1	−2.9 (3)
O3—C3—C4—C5	−167.13 (18)	C8—C7—O2—C1	176.33 (17)
C2—C3—C4—C5	−48.7 (2)	O1—C1—O2—C7	81.6 (2)
O4—C4—C5—O1	−65.9 (2)	C2—C1—O2—C7	−160.48 (16)
C3—C4—C5—O1	54.8 (2)	O7—C11—O3—C3	−1.2 (3)
O4—C4—C5—C6	53.2 (3)	C12—C11—O3—C3	178.36 (17)
C3—C4—C5—C6	173.9 (2)	C2—C3—O3—C11	154.73 (16)
O6—C9—C10—F2	−10.1 (3)	C4—C3—O3—C11	−83.1 (2)
N1—C9—C10—F2	170.94 (18)	O8—C13—O4—C4	−7.8 (3)
O6—C9—C10—F1	110.0 (2)	C14—C13—O4—C4	171.13 (17)
N1—C9—C10—F1	−69.0 (2)	C5—C4—O4—C13	−142.17 (18)
O6—C9—C10—F3	−130.8 (2)	C3—C4—O4—C13	97.2 (2)

### Hydrogen-bond geometry (Å, º)

**Table:**

*D*—H···*A*	*D*—H	H···*A*	*D*···*A*	*D*—H···*A*
N1—H1*A*···O6^i^	0.88	2.14	2.959 (2)	155
C3—H3···O6^i^	1.00	2.45	3.308 (3)	144
C6—H6*B*···F3^ii^	0.98	2.63	3.487 (3)	147
C8—H8*A*···O5^iii^	0.98	2.48	3.445 (3)	169
C8—H8*B*···O5^iv^	0.98	2.41	3.206 (3)	137
C8—H8*C*···O8^v^	0.98	2.64	3.443 (3)	139
C14—H14*A*···O8^iv^	0.98	2.38	3.292 (3)	155

Symmetry codes: (i) *x*−1, *y*, *z*; (ii) −*x*, *y*−1/2, −*z*+3/2; (iii) *x*+1/2, −*y*+1/2, −*z*+1; (iv) *x*+1, *y*, *z*; (v) −*x*+1/2, −*y*+1, *z*−1/2.

## References

[bb1] Alhassan, A.-B., McCutcheon, D. C., Zeller, M. & Norris, P. (2012). *J. Carbohydr. Chem.* **31**, 371–383.

[bb2] Bruker (2002). *SMART* Bruker AXS Inc, Madison, Wisconsin, USA.

[bb3] Bruker (2003). *SAINT-Plus.* Bruker AXS Inc, Madison, Wisconsin, USA.

[bb4] Hübschle, C. B., Sheldrick, G. M. & Dittrich, B. (2011). *J. Appl. Cryst.* **44**, 1281–1284. 10.1107/S0021889811043202 PMC3246833 22477785

[bb5] Macrae, C. F., Bruno, I. J., Chisholm, J. A., Edgington, P. R., McCabe, P., Pidcock, E., Rodriguez-Monge, L., Taylor, R., van de Streek, J. & Wood, P. A. (2008). *J. Appl. Cryst.* **41**, 466–470.

[bb6] Parsons, S., Flack, H. D. & Wagner, T. (2013). *Acta Cryst.* B**69**, 249–259. 10.1107/S2052519213010014 PMC3661305 23719469

[bb7] Rao, V. S. R., Qasba, P. K., Chandrasekaran, R. & Balaji, P. V. (1998). *Conformation of Carbohydrates* Amsterdam: Harwood Academic Publishers.

[bb8] Sheldrick, G. M. (2007). *SADABS* University of Göttingen, Germany.

[bb9] Sheldrick, G. M. (2008). *Acta Cryst.* A**64**, 112–122. 10.1107/S0108767307043930 18156677

[bb10] Westrip, S. P. (2010). *J. Appl. Cryst.* **43**, 920–925.

